# Plant Nucleolar Stress Response, a New Face in the NAC-Dependent Cellular Stress Responses

**DOI:** 10.3389/fpls.2017.02247

**Published:** 2018-01-09

**Authors:** Iwai Ohbayashi, Munetaka Sugiyama

**Affiliations:** ^1^FAFU-UCR Joint Center and Fujian Provincial Key Laboratory of Haixia Applied Plant Systems Biology, Haixia Institute of Science and Technology, Fujian Agriculture and Forestry University, Fuzhou, China; ^2^Botanical Gardens, Graduate School of Science, The University of Tokyo, Tokyo, Japan

**Keywords:** nucleolar stress response, nucleolus, NAC transcription factor, ribosome biogenesis, ribosomal protein, Pre-rRNA processing, cell proliferation, development

## Abstract

The nucleolus is the most prominent nuclear domain, where the core processes of ribosome biogenesis occur vigorously. All these processes are finely orchestrated by many nucleolar factors to build precisely ribosome particles. In animal cells, perturbations of ribosome biogenesis, mostly accompanied by structural disorders of the nucleolus, cause a kind of cellular stress to induce cell cycle arrest, senescence, or apoptosis, which is called nucleolar stress response. The best-characterized pathway of this stress response involves p53 and MDM2 as key players. p53 is a crucial transcription factor that functions in response to not only nucleolar stress but also other cellular stresses such as DNA damage stress. These cellular stresses release p53 from the inhibition by MDM2, an E3 ubiquitin ligase targeting p53, in various ways, which leads to p53-dependent activation of a set of genes. In plants, genetic impairments of ribosome biogenesis factors or ribosome components have been shown to cause characteristic phenotypes, including a narrow and pointed leaf shape, implying a common signaling pathway connecting ribosomal perturbations and certain aspects of growth and development. Unlike animals, however, plants have neither p53 nor MDM2 family proteins. Then the question arises whether plant cells have a nucleolar stress response pathway. In recent years, it has been reported that several members of the plant-specific transcription factor family NAC play critical roles in the pathways responsive to various cellular stresses. In this mini review, we outline the plant cellular stress response pathways involving NAC transcription factors with reference to the p53-MDM2-dependent pathways of animal cells, and discuss the possible involvement of a plant-unique, NAC-mediated pathway in the nucleolar stress response in plants.

The core processes of ribosome biogenesis, such as ribosomal RNA (rRNA) transcription, pre-rRNA processing, and ribosome assembly, take place in the nucleolus, which is the most prominent nuclear domain. These processes are finely controlled by many nucleolar factors, to build precisely small and large ribosome particles. Perturbations of any of the steps of ribosome biogenesis in the nucleolus cause a type of stress called nucleolar stress or ribosomal stress, which stimulates a specific signaling pathway in animal cells. Recent studies have implicated a plant-unique pathway in the nucleolar stress response of plant cells. In this mini review, we will discuss how plant cells sense and respond to nucleolar stress, with reference to the p53-dpeendent nucleolar stress response pathway of animal cells.

## Ribosome biogenesis in the nucleolus

The nucleolus is formed around the nuclear rDNA regions where tandem repeats of rRNA genes lie, and serves as a site for the main part of biogenesis of the ribosome, a huge ribonucleoprotein machinery that executes translation of the nucleotide sequence of mRNAs into the amino acid sequence of proteins. In the nucleolus, ribosome biogenesis starts with the transcription of pre-rRNAs from rRNA genes, followed by their processing and assembly with ribosomal proteins (RPs) into two ribosome subunits, the small subunit (SSU) and the large subunit (LSU).

Among the four species of rRNA, i.e., 5S, 5.8S, 18S, and 25–28S rRNAs, only 5S rRNA is transcribed separately, whereas the other three rRNAs are transcribed as a single precursor molecule. This precursor undergoes sequential processing events, such as base modification, cleavage, and trimming, which are tightly linked with the stepwise assembly of RPs onto pre-rRNAs. These events are guided by ribosome biogenesis factors (RBFs), which are enriched in the nucleolus, including small nucleolar RNAs (snoRNAs), snoRNA-associated proteins, and many other proteins. The processing pathway of pre-rRNAs and the repertoire of RBFs are partly different but largely common between yeast, animals, and plants (Henras et al., 2015; Weis et al., 2015). The nucleolar activities of pre-rRNA processing and RP assembly produce near-complete forms of the SSU and LSU. The SSU comprises 18S rRNA and 33 types of RPs, whereas the LSU comprises 5S, 5.8S, and 25–28S rRNAs and 46–48 types of RPs. The SSU and LSU RPs are highly conserved throughout eukaryotes, with only a few exceptions.

## Nucleolar stress response pathway in animal cells

The elaborately regulated biogenesis of ribosomes is disturbed under unhealthy conditions for various reasons, such as nutrient starvation, hypoxia, heat shock, chemical suppression of ribosome biogenesis, genetic impairments of RBFs, and deficiencies of RPs (Mayer and Grummt, 2005; Boulon et al., 2010). In animal cells, such perturbations of ribosome biogenesis, regardless of origin, are mostly accompanied by structural disorders of the nucleolus, although they are not always obvious, and cause a particular type of stress called nucleolar stress or ribosomal stress, which stimulates specific signaling pathways leading to cell-cycle arrest, senescence, or apoptosis (Olausson et al., 2012; James et al., 2014). These stress signaling pathways are classified into two types, p53-dependent and -independent ones (Olausson et al., 2012; James et al., 2014; Russo and Russo, 2017).

The canonical and most extensively studied pathway of animal nucleolar stress response is the p53-dependent pathway. The well-known antitumor transcription factor p53 and its destabilizer MDM2 play a pivotal role in this pathway. In normal conditions, the activity of p53 is suppressed to a low level under the control of MDM2, which acts as a component of E3 ubiquitin ligase and targets p53, leading to the ubiquitination and proteasomal degradation of p53. Upon nucleolar stress, RPs are released from the nucleolus into the nucleoplasm. Several of the RPs released, including RPL5, RPL11, RPL23, and RPS7, bind directly to MDM2 as effectors, thus preventing its action on p53 (Lohrum et al., 2003; Dai and Lu, 2004; Jin et al., 2004; Chen et al., 2007). As a result, p53 becomes stable and active in regulating the expression of genes that are involved in cell-cycle arrest, senescence, and apoptosis (Zhang and Lu, 2009; Deisenroth and Zhang, 2010; Golomb et al., 2014). Genes that are expressed at relatively high levels of p53 activity induce senescence or apoptosis, whereas genes that are expressed at low p53 levels induce cell-cycle arrest (Lai et al., 2007).

In recent years, increasing evidence has indicated that additional mechanisms not involving p53 participate in nucleolar stress response. For instance, RPs released from the nucleolus upon nucleolar stress, such as RPL11 and RPS14, were shown to repress the activity of the oncoprotein transcription factor c-Myc, which is crucial for the expression of many genes involved in cell growth and proliferation, through direct binding to the c-Myc protein and/or controlling microRNA-induced silencing complex (miRISC)-mediated turnover of *c-Myc* mRNA (Dai et al., 2007; Challagundla et al., 2011; Zhou et al., 2013). It was also reported that in response to nucleolar stress, released RPs can induce cell cycle arrest via regulation of the E2F transcription factor E2F-1 or CDK inhibitors p21^Waf1/Cip1^ and p27^Kip1^, independently of p53 (Iadevaia et al., 2010; Donati et al., 2011; Russo et al., 2013).

## Effects of the perturbation of ribosome biogenesis in plants

In plants, especially in the model plant Arabidopsis, many ribosome-related mutants, which are impaired in an RBF- or RP-encoding gene, have been isolated and characterized. Most of these mutants exhibit a similar spectrum of phenotypes, including a narrow and pointed leaf shape and retardation of root growth (Byrne, 2009; Horiguchi et al., 2012; Tsukaya et al., 2013; Weis et al., 2015). A severe loss of the adaxial–abaxial polarity of leaves under the genetic background of *asymmetric leaves 1* (*as1*) or *as2* is also a notable phenotype that is shared by the ribosome-related mutants (Pinon et al., 2008; Horiguchi et al., 2011; Huang et al., 2014; Matsumura et al., 2016). These common phenotypic features of various ribosome-related mutants imply the existence of a common mechanism that regulates growth and development in response to various perturbations of ribosome biogenesis.

At the subcellular level, structural changes of the nucleolus have been reported for various RBF mutants (Table 1). In most cases, enlargement of the nucleolus was observed in association with excessive accumulation of intermediates of pre-rRNA processing. This nucleolar enlargement is sometimes linked with the development of a nucleolar cavity, i.e., nucleolar vacuolation (Ohbayashi et al., 2011). Moreover, in the Arabidopsis mutant that carries a disrupted nucleolin gene (*AtNUC-L1*), nucleolar disorganization and decondensation of the rDNA chromatin structure were shown to co-occur (Pontvianne et al., 2007). These findings indicate that perturbation of rRNA biogenesis generally induces structural disorders of the nucleolus in plant cells, as well as in animal cells (Nishimura et al., 2015).

**Table 1:** List of Arabidopsis genes that have been shown to participate in the function and/or structural integrity of the nucleolus.

**Gene symbol**	**AGI code**	**Protein product**	**Protein localization**	**Mutant allele**	**Mutation type**	**Mutant effects on**			**Mutant suppression by *sriw1***	**References**
						**Nucleolar structure**	**Nucleolar functions**	**Growth and development**		
*RID2*	At5g57280	Methyltransferase	Nucleolus	*rid2-1*	Base substitution (missense)	Enlargement accompanied by vacuolation	Impairment of pre-rRNA processing	Temperature-dependent defects in leaf blade development and root growth in seedlings, and in callus formation, adventitious root formation and shoot regeneration in tissue culture, and severe loss of leaf abaxial–adaxial polarity in *as1* and *as2*	Yes	Konishi and Sugiyama, 2003; Ohbayashi et al., 2011, 2017; Shinohara et al., 2014; Matsumura et al., 2016
				*rid2-2, rid2-3*	T-DNA insertion	—	—	Female gametophyte lethality, maternal sterility, or maternal embryonic lethality	—	Ohbayashi et al., 2011
*RID3*	At3g49180	WD40 repeat protein	Nucleus	*rid3*	Base substitution (missense)	Enlargement	Impairment of pre-rRNA processing	Temperature-dependent defects in leaf blade development and root growth in seedlings, and in shoot regeneration in tissue culture	Yes	Tamaki et al., 2009; Shinohara et al., 2014; Ohbayashi et al., 2017
*RH10*	At5g60990	DEAD-box RNA helicase	Nucleolus	*rh10-1*	Base substitution (missense)	Enlargement	Impairment of pre-rRNA processing	Temperature-dependent defects in leaf blade development and severe loss of leaf abaxial–adaxial polarity in *as1* and *as2*	Yes	Matsumura et al., 2016; Ohbayashi et al., 2017
*ATNUC-L1*	At1g48920	Nucleolin	Nucleolus	*Atnuc-L1-1*	T-DNA insertion	Disorganization (rDNA heterochromatin decondensation)	Impairment of pre-rRNA processing (blockage of cleavage at least at the P site) and increased transcription of specific rRNA variants	Pleiotropic defects in growth and development, including abnormal leaf morphogenesis, and severe loss of leaf abaxial–adaxial polarity in *as1* and *as2*	—	Pontvianne et al., 2007, 2010; Matsumura et al., 2016
				*parl1-1*	2 bp insertion	—	—	Abnormal vein patterning, pointed narrow leaf, retarded root growth, and reduced fertility	—	Petricka and Nelson, 2007
				*parl1-2* (*Atnuc-L1-2*)	T-DNA insertion	Disorganization (rDNA heterochromatin decondensation)	Impairment of pre-rRNA processing and increased transcription of specific rRNA variants	Abnormal vein patterning, pointed narrow leaf, retarded root growth, reduced fertility, and severe loss of leaf abaxial–adaxial polarity in *as1* and *as2*	—	Petricka and Nelson, 2007; Pontvianne et al., 2010; Matsumura et al., 2016
*DOMINO1*	At5g62440	Small plant-specific protein	Nucleus	*domino1*	T-DNA insertion	Enlargement	Inactivation of ribosome biogenesis	Retarded embryogenesis	—	Lahmy et al., 2004
*NOF1*	At1g17690	DUF1253-containing protein	Nucleolus	*nof1-1*	T-DNA insertion (in the promoter region)	Enlargement	Increase in rRNA transcription	Embryonic lethality (irregular pattern and/or additional cell division, and lack of cell adhesion)	—	Harscoët et al., 2010
				*nof1-2*	T-DNA insertion	—	—	Female gametophyte lethality	—	Harscoët et al., 2010
*APUM23*	At1g72320	Pumilio protein	Nucleolus	*apum23-1, apum23-2*	T-DNA insertion	Enlargement	Impairment of processing/degradation of pre-rRNAs	Pleiotropic defects in growth and development, including abnormal leaf morphogenesis	—	Abbasi et al., 2010
				*apum23-3*	Base substitution (missense)	—	Impairment of pre-rRNA processing	Pleiotropic defects in growth and development, including abnormal leaf morphogenesis and severe loss of leaf abaxial–adaxial polarity in *as1* and *as2*	—	Huang et al., 2014
*TOZ*	At5g16750	WD40 repeat protein	Nucleolus	*toz*	Ds insertion	Enlargement	Impairment of pre-rRNA processing	Embryonic lethality (aberrant cell division)	—	Griffith et al., 2007
*ATLa1*	At4g32720	La motif protein	Nucleoplasm and nucleolar cavity (?)	*atla1-1, atla1-2*	T-DNA insertion	Enlargement	Interference with ribosome biogenesis (?)	Embryonic lethality (arrest at the early globular stage)	—	Fleurdépine et al., 2007
*ATREN1*	At1g77570	Heat-shock transcription factor	Nucleolus	*atren1*	T-DNA insertion	Enlargement	Interference with ribosome biogenesis (?)	Defects in male gametophyte development	—	Renák et al., 2014

Taking together the morphological and cytological characteristics of ribosome-related mutants, we can speculate that perturbations of ribosome biogenesis and the resultant structural disorders of the nucleolus affect several aspects of plant growth and development via a specific signaling pathway. If this is true, then what is the pathway? By analogy with animal cells, it seems reasonable to assume the presence of a nucleolar stress response pathway at work in plant cells. However, as plants have no homologs of p53 or MDM2 (Huart and Hupp, 2013), the plant pathway should not involve a p53- and MDM2-dependent mechanism and might be distinct from the nucleolar stress response pathway of animal cells.

## Implication of ANAC082 as a mediator of the nucleolar stress response in plants

Very recently, the important evidence of the nucleolar stress response pathway in plants came from molecular genetic studies of Arabidopsis mutants, *root initiation defective 2* (*rid2*) and *suppressor of rid two 1* (*sriw1*). *rid2* is a temperature-sensitive mutant that is impaired in pre-rRNA processing because of a missense mutation in the gene encoding a putative RNA methyltransferase and is characterized phenotypically by severe defects in cell proliferation and a striking enlargement and vacuolation of the nucleolus at high temperatures (Ohbayashi et al., 2011). *sriw1* is a *rid2* suppressor mutant that was isolated from a mutagenized population of *rid2* (Ohbayashi et al., 2017). The *sriw1* mutation markedly alleviated the cell proliferation defects of *rid2* without relieving the impaired pre-rRNA processing. It was further shown that the *sriw1* mutation had the abilities to restore growth and development not only in *rid2* but also generally in various ribosome-related mutants, including both RBF mutants and RP mutants, and to confer weak resistance to chemicals that interfere with ribosome biogenesis or ribosomal function. *sriw1* was identified as a loss-of-function mutation of the gene encoding ANAC082, which belongs to the plant-specific transcription factor family NAM/ATAF/CUC (NAC). The expression level of *ANAC082* was temperature-dependently increased in temperature-sensitive RBF mutants. These findings collectively implicate ANAC082 as a key mediator that works downstream of perturbations of ribosome biogenesis and nucleolar disorders, thus leading to growth defects and developmental alterations in plants; i.e., plant cells are considered to respond to nucleolar stress via a plant-unique signaling pathway mediated by ANAC082.

## Possible mechanisms of sensing nucleolar stress in plants

To understand the nucleolar stress response in plants, how plant cells sense perturbed ribosome biogenesis and nucleolar disorders is one of the most critical questions. Among the fragmentary pieces of information available currently regarding the plant pathway of the nucleolar stress response, a clue to one of its early steps can be found in the elevated expression of *ANAC082* in RBF mutants (Ohbayashi et al., 2017). Transcriptional regulation and/or post-transcriptional regulation may contribute to this upregulation of *ANAC082* expression. With respect to post-transcriptional regulation, the existence of a conserved upstream open reading frame (uORF) in the upstream region of the main ORF in the *ANAC082* mRNA is of considerable note (Takahashi et al., 2012). This uORF was demonstrated to have an amino-acid-sequence-dependent, negative effect on the expression of the downstream main ORF (Ebina et al., 2015). Most of the regulatory uORFs studied to date cause ribosome stalling at their termination codon, which impedes the access of ribosomes to the main ORF and often induces nonsense-mediated mRNA decay (Gao and Geballe, 1996; Law et al., 2001; Gaba et al., 2005; Uchiyama-Kadokura et al., 2014). Such uORF-dependent control can be affected by ribosomal defects, as was reported for the expression of uORF-containing genes that encode auxin signaling factors in the Arabidopsis RP mutant *rpl24b* (Nishimura et al., 2004, 2005; Zhou et al., 2010). Taking these findings into consideration, the uORF of *ANAC082* might be a candidate site of nucleolar stress sensing. A possible underlying mechanism is that, under nucleolar stress, because of a shortage of functional ribosomes, imbalance of ribosomal subunits, or some other abnormal situation of ribosomes, the constraint of *ANAC082* expression by ribosome stalling at the uORF is loosened, resulting in the activation of ANAC082.

It is also possible that the activity of ANAC082 is regulated via a protein–protein interaction upon nucleolar stress, given the knowledge that NAC transcription factors generally form a homodimer or heterodimer through interaction at the N-terminal NAC domain (Ernst et al., 2004). ANAC082 has a potential of binding to several species of NAC transcription factors, including NAC1, CUP-SHAPED COTYLEDON 2 (CUC2), ANAC103, and VASCULAR-RELATED NAC-DOMAIN (VND) proteins (Yamaguchi et al., 2015). Moreover, ANAC082 is part of the list of transcription factors that can bind to the WWE domain-containing, non-NAC proteins RADICAL-INDUCED CELL DEATH 1 (RCD1), and SIMILLAR TO RCD-ONE 1 (SRO1) (Jaspers et al., 2009). If some of these proteins act as a partner of ANAC082, it is also possible that the partner, instead of ANAC082, is responsible for the process of nucleolar stress sensing.

## Multiple NACs in plant cellular stress responses corresponding to multiple roles of p53 in animal cellular stress responses

In animal cells, p53 participates as a nodal regulator not only in the nucleolar stress response pathway but also in pathways that respond to several other cellular stresses, such as oncogenic stress, DNA damage stress, and oxidative stress (Serrano et al., 1997; Horn and Vousden, 2007; Hu et al., 2012). Oncogenic stress, which is caused by the inappropriate expression of oncogenes or proto-oncogenes, is transduced to the activation of p53 mainly through the transcriptional upregulation and protein stabilization of the tumor suppressor p14^ARF^ and the consequent inhibition of MDM2 activity by p14^ARF^ (Sherr and Weber, 2000; Gallagher et al., 2006; Chen et al., 2010). Another route connecting oncogenic stress to p53 activation depends on the binding of the 5S ribonucleoprotein complex (5S RNP), consisting of 5S rRNA, RPL5, and RPL11, to MDM2 (Nishimura et al., 2015). In the pathway of DNA damage stress response, sensing DNA double-strand breaks, persisting single-stranded DNA, or stalled replication forks, the PI3K-related protein kinase ATM or ATR is activated and phosphorylates specific sites of p53 and MDM2, which results in the activation and stabilization of p53 (Shiloh, 2001; Maréchal and Zou, 2013). Oxidative stress caused by reactive oxygen species (ROS) induces the activation of p53 via the DNA damage stress response pathway, which is triggered by oxidation damage of DNA and via direct activation of ATM by oxidation (Barzilai and Yamamoto, 2004; Guo et al., 2010). Importantly, the ROS-induced p53 signaling acts toward generating ROS through the downstream effector p66^shc^, which comprises a positive feedback loop (Migliaccio et al., 1999; Nemoto and Finkel, 2002).

Among the cellular stresses with responses that rely on p53 in animal cells, DNA damage stress and oxidative stress also occur in plant cells. Therefore, as is the case for the nucleolar stress response, the question arises regarding how plant cells respond to these stresses in the absence of p53 homologs. In Arabidopsis, it was demonstrated by molecular genetic studies that the DNA damage stress response is governed by SUPPRESSOR OF GAMMA RESPONSE 1 (SOG1)/ANAC008, a transcription factor of the NAC family (Preuss and Britt, 2003; Yoshiyama et al., 2009). Upon DNA damage stress, the SOG1 protein is hyperphosphorylated in an ATM-dependent manner and thereby activated to regulate gene expression, which resembles the regulatory mechanism of p53 by ATM and ATR in animal cells (Yoshiyama et al., 2013). Based on these findings, SOG1 is often discussed as a functional counterpart of p53, although SOG1, and p53 have no sequence similarity (Yoshiyama, 2015). Oxidative stress induces DNA damage and elicits the DNA damage stress response in plant cells, as well as in animal cells. Moreover, in plants, oxidative stress promotes leaf senescence independently of DNA damage. Several NAC transcription factors, such as ARABIDOPSIS THALIANA ACTIVATION FACTOR 1 (ATAF1)/ANAC002 and ANAC092, have been reported as mediators of this process (Balazadeh et al., 2010; Garapati et al., 2015). Of these, the most notable one is the membrane-bound NAC protein NAC WITH TRANSMEMBRANE MOTIF 1-LIKE 4 (NTL4)/ANAC053, as this transcription factor is activated through oxidative proteolysis by ROS and promotes ROS production, thus representing a positive feedback loop, similar to that of the p53-dependent oxidative stress response pathway in animal cells (Lee et al., 2012, 2014).

**Figure 1:**
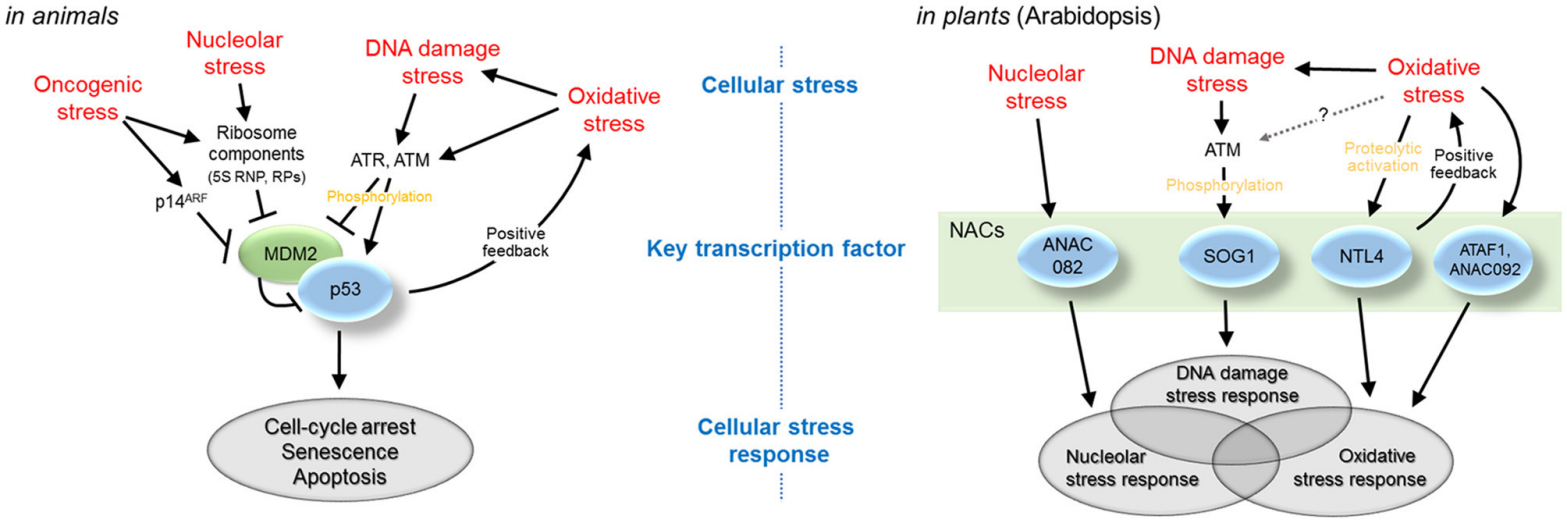
NAC-dependent pathways of plant cellular stress responses and p53-dependent pathways of animal cellular stress responses. Animal cells utilize p53 to regulate responses to nucleolar stress, DNA damage stress, and oxidative stress, while plant cells employ multiple NAC transcription factors for these roles of p53.

In summary, in the context of cellular stress responses, animal cells utilize p53 to regulate responses to nucleolar stress, DNA damage stress, and oxidative stress, while plant cells employ multiple NAC transcription factors for these roles of p53 (Figure 1). Cellular stresses such as nucleolar stress, DNA damage stress, and oxidative stress are both extrinsic and intrinsic to basic cellular activities of ribosome biogenesis, genome replication, and energy metabolism; thus, they are unavoidable in all organisms. To sense and cope with cellular stresses, animals have evolved signaling systems that share a limited number of transcription factors, including p53, as central regulators. In contrast, plants have evolved stress signaling systems that use different transcription factors for different stresses. This strategy seems related to the diversification of transcription factors of particular groups in plants. In this respect, the NAC family has received increasing attention. NAC is one of the largest families of plant transcription factors and includes more than 100 members (Zhu et al., 2012). Many of the NAC-family members have been previously implicated in responses to abiotic and biotic stresses triggered by external causes, such as drought, salt, cold, and pathogen infection (Nakashima et al., 2012; Puranik et al., 2012; Nuruzzaman et al., 2013). Recently discovered NAC-dependent pathways of intrinsic cellular stress responses have added more emphasis to the variety of the NAC transcription factors in plant stress responses.

## Future perspectives

In conclusion, recent studies collectively suggest that plant cells use a specific pathway for nucleolar stress response involving ANAC082, a member of the plant-unique transcription factor family NAC. Considering the possible role of uORF in the regulation of *ANAC082* expression, the molecular mechanism of this pathway may be quite different from that of the p53-dependent pathway of nucleolar stress response in animal cells; nevertheless, ANAC082 might be regarded as a counterpart of p53 because of their common role as critical transcriptional regulators of the nucleolar stress response. During plant evolution, NAC transcription factors have been highly diversified, and different NAC factors have been assigned to different pathways of stress responses in plants, several of which correspond to the different roles of p53 in the stress responses of animal cells. In this sense, ANAC082 might also be considered a player of the NAC team that is responsible for the tasks carried out by p53 in animal cells.

The ANAC082-dependent pathway underlying the plant nucleolar stress response is a recent concept, and its molecular details remain totally unknown. A particularly important issue is the elucidation of how nucleolar stress is sensed in plants to activate the ANAC082 pathway. Further studies focusing on this problem may lead to the understanding of nucleolar surveillance in plant cells and open new horizons in plant nucleolar biology.

## Author contributions

IO and MS conceived this review and wrote the manuscript.

### Conflict of interest statement

The authors declare that the research was conducted in the absence of any commercial or financial relationships that could be construed as a potential conflict of interest.
